# Research on decoupled air speed and air volume adjustment methods for air-assisted spraying in orchards

**DOI:** 10.3389/fpls.2023.1250773

**Published:** 2023-09-08

**Authors:** Hanjie Dou, Changyuan Zhai, Yanlong Zhang, Liping Chen, Chenchen Gu, Shuo Yang

**Affiliations:** ^1^ Intelligent Equipment Research Center, Beijing Academy of Agriculture and Forestry Sciences, Beijing, China; ^2^ National Engineering Research Center of Intelligent Equipment for Agriculture, Beijing, China; ^3^ National Engineering Research Center for Information Technology in Agriculture, Beijing, China

**Keywords:** orchard, precision spraying, air speed, air volume, decoupled control

## Abstract

Different fruit tree canopies have different requirements for air speed and air volume. Due to the strong relationship between air speed and air volume, the decoupled control of air speed and air volume cannot be achieved using the existing sprayers. In this study, an innovative air-assisted sprayer that supports the independent adjustment of fan speed (0-2940 r/min) and air outlet area (1022.05-2248.51 cm^2^) is developed, and the maximum air speed and air volume of the sprayer outlet are 45.98 m/s and 37239.94 m^3^/h, respectively. An independent adjustment test of the fan speed and air outlet area was carried out. The results indicated that the fan speed and air outlet area have opposing adjustment effects on air speed and air volume; decoupled control of the outlet air speed and air volume can thus be achieved through combined control of the fan speed and air outlet area. A test was carried out on combined fan speed and air outlet area control. Two decoupled air speed and air volume adjustment models were established, one with a constant air speed and variable air volume and the other with a constant air volume and variable air speed. The test results show that the air volume adjustment model with constant air speed had a maximum mean error of 1.13%, and the air speed adjustment model with constant air volume had a maximum mean error of 1.67%. The results will provide theoretical and methodological support for the development of airflow adjustment systems for orchard air-assisted sprayer.

## 1 Introduction

The global fruit industry has become the third-largest agricultural planting industry after the grain and vegetable industries. By 2020, the area of orchards in China had reached 1.33×10^7^ hm^2^ (Zheng et al., 2020; Wei et al., 2023), ranking first in the world. Orchard pest control has long relied on chemical pesticides. According to statistics, 66-90% of fruit crops can be lost without pesticides (Mahmud et al., 2021b). The application rate of pesticides per unit area in China is considerably higher than the world average. Excessive application of pesticides not only wastes pesticides and causes environmental pollution but also greatly threatens the safety of agricultural products (Gil et al., 2014; Manandhar et al., 2020; Zheng and Xu, 2021). To improve the pesticide use efficiency, orchard precision spraying technology has gradually emerged in recent years. Orchard precision spraying refers to the target-oriented variable-rate application of pesticides through the online detection of the target characteristics and disease characteristics of orchards with sensor systems, the calculation of the pesticide dose and airflow requirements for air-assisted spraying according to the detected characteristics, and the variable adjustment of the pesticide dose and airflow supply (He, 2020).

Orchard air-assisted precision spraying requires on-demand pesticide dose control technology and on-demand airflow adjustment technology. Scholars worldwide have conducted considerable research regarding on-demand pesticide dose control, explored target orchard spraying control technology (Salcedo et al., 2021; Warneke et al., 2021; Dou et al., 2022) and developed variable adjustment models and methods (such as pipe and nozzle flow or the direct-injection flow of liquid pesticides) based on pressure, travel speed and pulse width modulation (PWM) to potentially solve the key theoretical and methodological problems of dose demand-based variable spraying control (Shen et al., 2017; Grella et al., 2022). However, there are few related studies of the on-demand control of airflow. Airflow includes three elements: air speed, air volume and airflow direction. For the current airflow adjustment devices, the airflow is mainly adjusted by changing the fan speed, the air inlet area, the air outlet area, the inclination angle of the deflector and the inclination angle of the air outlet (Zhai et al., 2018a). The fan speed, air inlet area, and air outlet area can be adjusted by changing the air speed and air volume at the air outlet. Professor Lander at Cornell University in the United States started research on airflow adjustment technology as early as 2010. By adding louvres at the air outlet of an air-assisted sprayer to change the airflow and integrating infrared target technology into the device, Landers (2010) and Khot et al. (2012) developed a sprayer that can control air speed and air volume according to the profile information of the fruit tree canopy and found that the use of air louvres resulted in a 30% increase in deposition and a 75% reduction in drift during trials in apple orchards. Qiu et al. (2016) adjusted the airflow by changing the fan speed to study the effects of airflow on deposition and drift. Hołownicki et al. (2017) designed a variable air volume system with a variable fan blade angle and fan speed and developed a target orchard sprayer with an adjustable air volume. Doruchowski et al. (2011a) ; Doruchowski et al. (2011b) successfully adjusted the air speed and air volume by changing the openings of the air inlet and outlet. Li et al. (2017) designed an orchard air-assisted sprayer for which the air volume can be adjusted according to an empirical parameter formula. Mahmud et al. (2021a); Mahmud et al. (2022) designed an orchard air-assisted sprayer with a variable air inlet area, used light detection and ranging (LiDAR) to obtain characteristic information from the fruit tree canopy, established an equation describing the relationship between the laser point cloud and the airflow demand, and realized online airflow adjustment. The inclination angles of the deflector and air outlet mainly influence the airflow direction, and Pai et al. (2009) successfully adjusted the outlet airflow direction by changing the inclination angle of the deflector. Osterman et al. (2013) designed a LiDAR-based orchard air-assisted sprayer with variable dimensions, and the position of the spray arm could be adjusted in real time according to the canopy feature information obtained by LiDAR; thus, the position of the air outlet of the bellows could be changed to achieve airflow adjustment at different canopy positions. Jiang et al. (2020) designed an air volume control system for an orchard multipipe air sprayer by installing a butterfly valve at each air outlet duct position to control the backflow ratio of the outlet air volume; they experimentally demonstrated that spraying with variable air volumes can improve canopy deposition by 17.3%, reduce deposition below the canopy and in gaps by 21.6% and 40.7%, respectively, and reduce airborne drift by 50.9%. Xu et al. (2013) and Zhai et al. (2018b) designed an orchard air-assisted sprayer with an adjustable spray height, and airflow adjustment was achieved by changing the inclination angle of the bellows.

In summary, most existing airflow adjustment devices only have a single adjustment mode, but due to the coupled relationship between the adjustment of air speed and air volume, these devices fail to provide independent adjustments for air speed and air volume. Different fruit trees require different combinations of air speed and air volume. In orchard air-assisted spraying, there are obvious differences in the canopy in different growth periods and for types of fruit trees. To improve the spray deposition in the canopy and reduce the spray drift in nontarget areas, differentiated canopies have different requirements for air speed and air volume at the sprayer outlet. For example, a canopy with dense branches and leaves but a small volume generally requires a high air speed and a low air volume, whereas a canopy with sparse branches and leaves but a large volume requires a low air speed and a high air volume. Therefore, the on-demand adjustment of airflow requires the decoupled control of air speed and air volume, which requires the online detection and calculation of the air speed and air volume demands of fruit trees to control the air supply actuator in real time and select the appropriate airflow, such that after the airflow loss in the conveying space, the pesticide can be delivered into the fruit tree canopy with the correct amount of airflow.

The objective of this study was to propose a method for the decoupled control of air speed and air volume at the sprayer outlet, and it could meet the air speed and air volume requirements of fruit trees with different combinations of canopy volume and branch/leaf density. Given that the independent adjustment of air speed and air volume cannot be achieved using the existing single-control-mode airflow adjustment devices, we designed an innovative airflow adjustment sprayer that supports the independent adjustment of the fan speed and air outlet area. Based on this sprayer, we carried out airflow adjustment experiments and obtained the variation characteristics of air speed and air volume at the air outlet under two independent adjustment modes. Based on the opposing adjustment effects of fan speed and air outlet area on air speed and air volume, we proposed a method for the decoupled control of the outlet air speed and air volume of the bellows. This study will provide theoretical and methodological support for research on and the development of airflow adjustment sprayers for orchards.

## 2 Materials and methods

### 2.1 Design of an air-assisted sprayer for orchards

**Figure 1:**
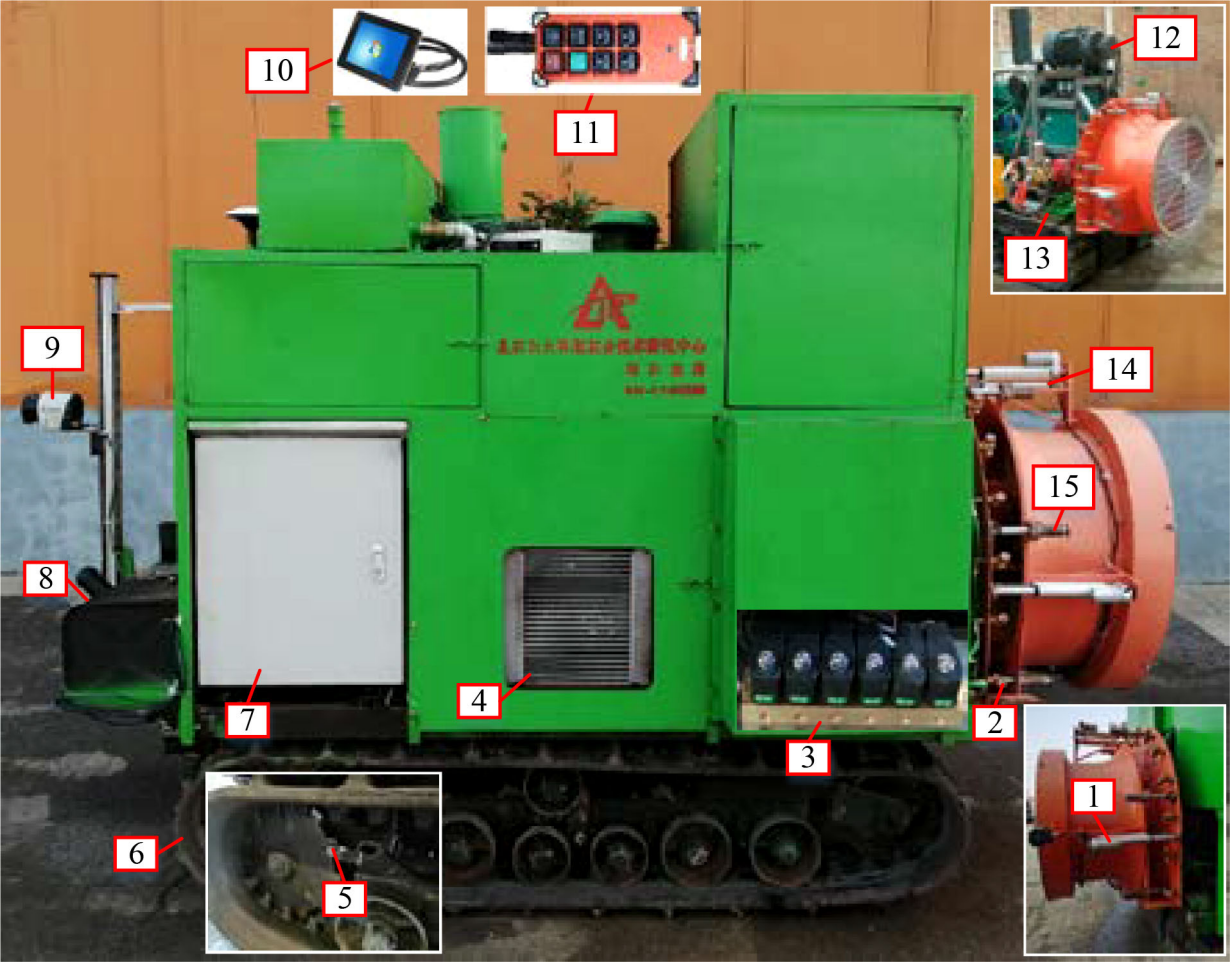
Orchard air-assisted sprayer that supports the independent adjustment of fan speed and air outlet area.

To realize the independent adjustment of fan speed and air outlet area and to facilitate subsequent research on methods for the decoupled control of air speed and air volume, an airflow adjustment sprayer was designed, as shown in **Figure 1**. The sprayer mainly consists of a fan speed adjustment device, an air outlet adjustment device, a spray system, LiDAR (LMS10100, SICK Ltd, Germany), a speed measuring device, a power system, a control system and a crawler chassis. The sprayer fan (CSF-660, Tianjin Cheng En Technology Co., Ltd, China) is an axial flow fan commonly used for orchard air-assisted spraying. The fan is connected to an AC motor (YE2-160M2-2, Tianjin Jin Rong Electromechanical Co., Ltd, China) by a belt, and the fan speed can be controlled from 0 to 2940 r/min by adjusting the motor speed. The back plate of the fan is connected to the air duct through six sliding bearings. The sliding bearings not only support the weight of the air duct but also allow relative movement between the bearing nut fixed on the air duct and the back plate. A linear actuator (12-150-50, Leicester Nuo (Dongtai) Transmission Machinery Manufacturing Co., Ltd, China) and a displacement sensor (KPM18, Shenzhen Milang Technology Co., Ltd, China) are installed between the back plate and the air duct. The movement of the linear actuator causes the sliding bearings to move, which in turn causes the air duct to move, such that the opening of the air outlet changes from 5 to 11 cm, corresponding to an air outlet area variation ranging from 1022.05 to 2248.51 cm^2^. The spray system is used for pesticide application. To facilitate the subsequent on-demand control of the pesticide dose, a pressure regulation device and a PWM control solenoid valve (YCH41, Yuyao yongchuang solenoid valve co., ltd, China) are added to the system to support the adjustment of the system spraying pressure and single-nozzle flow. LiDAR can obtain information on fruit tree canopy features (position, volume, leaf area density, etc.) in real time to provide a basis for the online control of the airflow and pesticide dose. The control system is used to control the movement of the crawler chassis, the operation of the spraying system and the adjustment of the fan speed and air outlet area, thus ensuring the stable operation of the test platform in accordance with the control requirements.

1. Linear actuator 2. Nozzle 3. Solenoid valve 4. Radiator 5. Speed sensor 6. Track chassis 7. Control cabinet 8. Fuel tank 9. LiDAR 10. PC 11. Remote control unit 12. Fan-driven motor 13. Spraying system 14. Displacement sensor 15. Slide bearing

### 2.2 Checking the airflow adjustment system

#### 2.2.1 Fan speed adjustment

**Figure 2:**
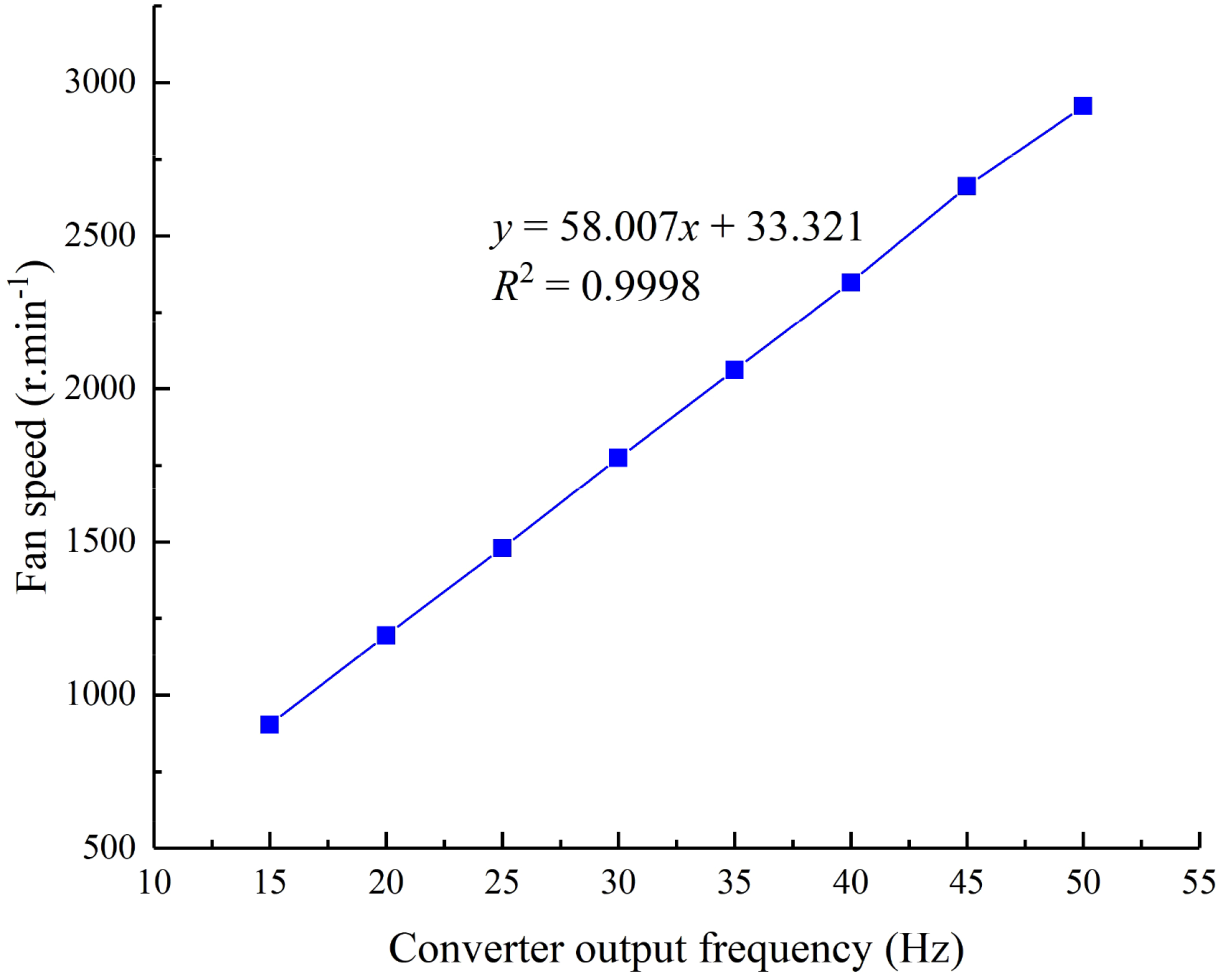
Relationship between the converter output frequency and fan speed.

To accurately control the fan speed, it is necessary to clarify the relationship between the converter output frequency and fan speed. The converter output frequency was set to 15, 20, 25, 30, 35, 40 or 45 Hz, and the fan speed was measured with a noncontact laser induction tachometer (TM680, Mitutoyo, Japan) at the connection between the fan and the AC motor. The recorded data for the converter output frequency and fan speed were fitted to obtain the corresponding relationship equation, as shown in **Figure 2**. The maximum fan speed was 2923 r/min, the *R^2^* of the equation was 0.9998, and the linearity between the converter output frequency and the fan speed was good; thus, the control requirements were met.

#### 2.2.2 Air outlet area adjustment

**Figure 3:**
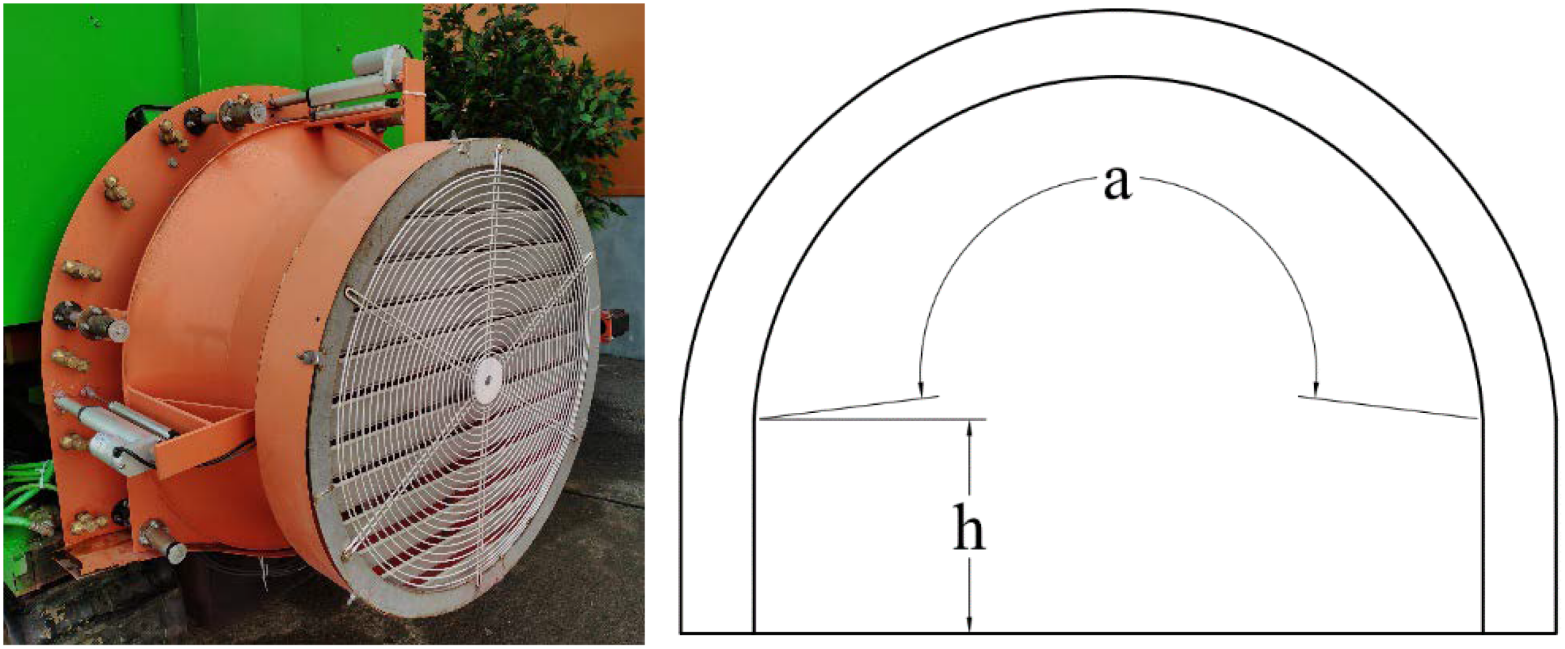
Schematic diagram of the air outlet area calculation.


The fan outlet is a ring structure, and the air outlet area is equivalent to the sum of the areas of two rectangles and one arc. The air outlet area changes with the size of the opening, as shown in **Figure 3**. The air outlet area is calculated with Formula (1).

(1) $$S_{OUT}=2hl+\frac{\pi dal}{360}$$

where *S*_OUT_ is the air outlet area of the fan (cm^2^), *l* is the size of the opening of the air outlet (cm), *h* is the height of the rectangular area of the air outlet (cm, set to 23.5 cm), *d* is the diameter of the arc area of the air outlet (cm, set to 90 cm), and *a* is the arc angle of the arc area of the air outlet (in degrees, set to 200.52°).

**Figure 4:**
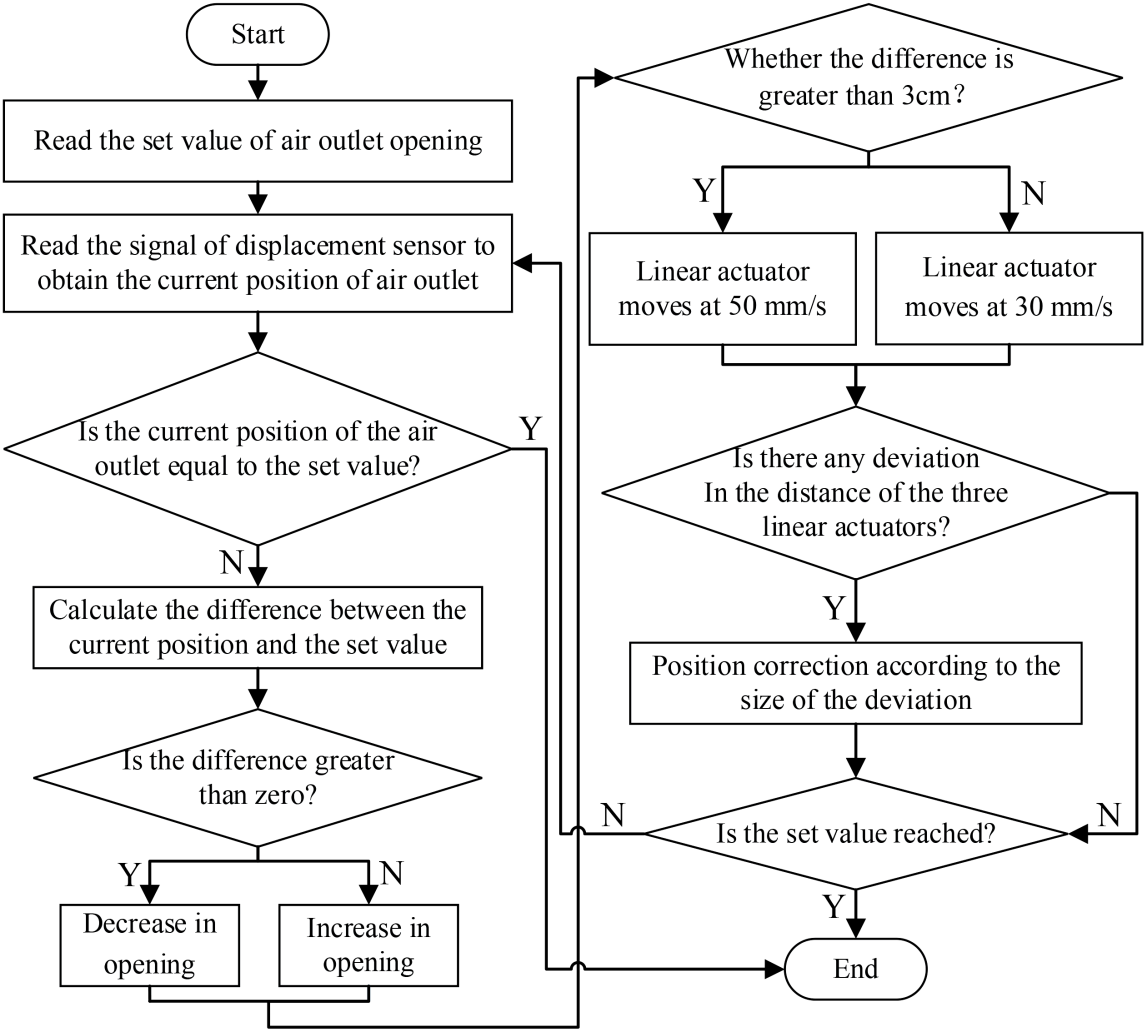
Flow chart of air outlet area adjustment.

The air outlet adjustment device is controlled by three linear actuators, and the maximum moving speed is 80 mm/s. During the adjustment process, if the moving distances of the linear actuators are not synchronized, the air outlet will be inclined, which affects the distribution of air speed and air volume at the air outlet. Additionally, a large displacement deviation of the three linear actuators may cause deformation of the fan air duct or damage to the linear actuators. To reduce the synchronization error in the adjustment process, differential control is introduced to the control program, the displacements of the linear actuators are monitored in real time by the displacement sensor, and the deviation is corrected. The flow chart of air outlet area adjustment is shown in **Figure 4**. Due to the influence of the weight of the fan, the middle position of the fan is directed slightly downwards. When adjusting the system, the moving speed of the middle linear actuator is set to be 5 mm/s more than that of the linear actuators on the left and right sides. Overall, the adjustment error of the air outlet opening varies within the range of 5-8 mm.

### 2.3 Pneumatic conveying system adjustment test

#### 2.3.1 Measurement of air speed at the fan outlet

**Figure 5:**
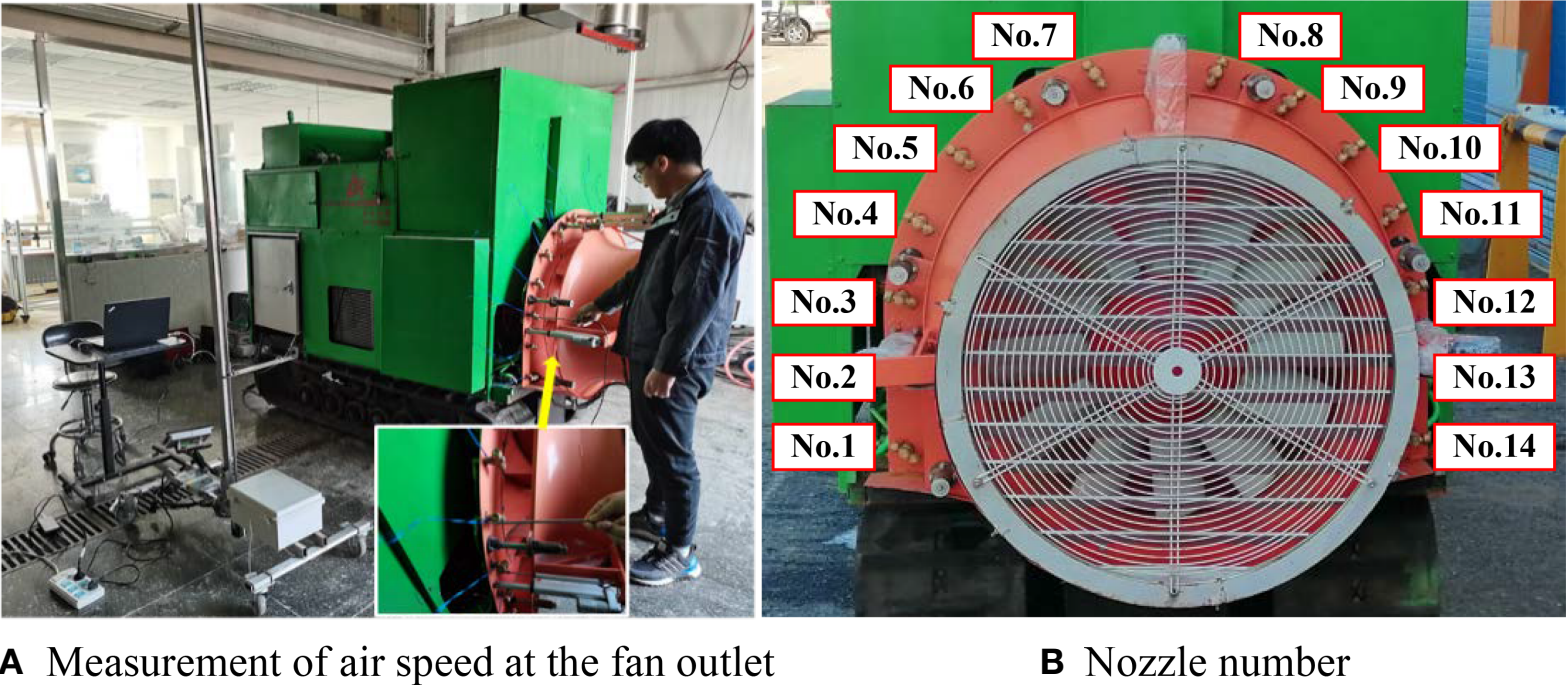
Air speed measurement for fan outlet **(A)** Measurement of air speed at the fan outlet **(B)** Nozzle number.

Because the nozzles at the fan outlet are positioned at equal intervals, one air speed measurement point is set at each nozzle position at the fan outlet, and the centre of the air velocity transducer probe is located at the outermost edge of the nozzle. A soft blue ribbon of a certain length is used at each nozzle position to determine the airflow direction at that position (Hoheisel et al., 2021), and this information is used to measure the air speed at each nozzle position with an air velocity transducer (8455-300, TSI, USA), as shown in **Figure 5**. The air velocity transducer is characterized by a speed measurement range of 0-50 m/s, an airflow response time of 0.2 s, and a measurement error of ±2%.

#### 2.3.2 Tests of the airflow variation characteristics

To obtain the airflow variation characteristics at the fan outlet during the independent control of fan speed and outlet area, a test of the airflow variation characteristics of the pneumatic conveying system under independent adjustment was designed. In the test of the independent adjustment of fan speed, the air outlet area was set to 2248.51 cm^2^, the converter output frequency of the fan drive motor was set in the range of 15-50 Hz, and the fan speed was adjusted once every 5 Hz. In the test of the independent adjustment of the air outlet area, the air speed was set to 936 r/min, the linear actuator was set in the range of 5-11 cm, and the air outlet area was adjusted once every 1 cm. The air speed measurement method shown in **Figure 5** was used to measure the air speeds at the 14 nozzle positions at the fan outlet. Each test was repeated three times, and the test data were recorded.

#### 2.3.3 Tests of the combined control of fan speed and air outlet area

To achieve changes in the outlet air speed and air volume under the combined control of fan speed and outlet area, decoupled air speed and air volume adjustment models were established, and a test for the combined control of fan speed and air outlet area was designed. During the test, the fan speed and the outlet area were controlled with the airflow adjustment sprayer, and variations within the ranges shown in **Table 1** were achieved. The air speed at the outlet was measured using the air speed measurement method shown in **Figure 5**. Each combination of parameters was tested three times, and a total of 49 tests were conducted to obtain 2058 air speed data points. The average value of the three test results at each nozzle position was used as the final air speed at that position. The average value of the air speed at each nozzle position was multiplied by the air outlet area to obtain the outlet air volume.

**Table 1:** Combined adjusted test parameters for the fan speed and air outlet area.

Adjusted parameters	Adjusted values						
Fan speed (r.min^-1^)	904	1195	1480	1775	2062	2348	2662
Air outlet area (cm^2^)	1022.05	1226.46	1430.87	1635.28	1839.69	2044.10	2248.51

#### 2.3.4 Verification tests of the decoupled air speed and air volume adjustment models

For the established air volume adjustment model with constant air speed and the air speed adjustment model with constant air volume, model verification tests were designed, and the test parameters are shown in **Table 2**. In the verification test of the air volume adjustment model with constant air speed, for each constant air speed value, five groups of air volume values were randomly selected within the allowable adjustment range of the air volume, and the fan speed and the air outlet area were calculated. The fan speed and the air outlet area were adjusted with the airflow adjustment sprayer. The outlet air speed under each combination of fan speed and air outlet area was measured and multiplied by the air outlet area at the corresponding position to obtain the air volume. The calculated air volume was compared with the actual measured air volume to obtain the adjustment error of the model under different combinations of parameter values. Using the same method, a verification test for the air speed adjustment model with a constant air volume was performed.

**Table 2:** Parameters of the validation test for the decoupled air speed and air volume adjusment models.

Adjustment model	Constant values of the air speed (m.s^-1^)	Values of the air volume (m^3^.h^-1^)				
Variable air volume with constant air speed	15.66	8200.00	9800.00	11000.00	12000.00	12500.00
	19.53	9000.00	10000.00	12000.00	14000.00	15000.00
	23.39	10000.00	13000.00	14000.00	16000.00	18000.00
	27.25	11000.00	13000.00	14000.00	16000.00	19000.00
	31.11	12000.00	13000.00	14000.00	16000.00	19000.00
	34.98	15000.00	18000.00	20000.00	24000.00	26000.00
	38.84	16000.00	19000.00	22000.00	24000.00	26000.00
Adjustment model	Constant values of the air volume (m^3^.h^-1^)	Values of the air speed (m.s^-1^)				
Variable air speed with constant air volume	8012.50	13.00	14.00	16.00	18.00	21.00
	11325.00	16.00	17.00	19.00	21.00	23.00
	14637.50	20.00	22.00	25.00	30.00	35.00
	17950.00	24.00	26.00	30.00	34.00	40.00
	21262.50	27.00	29.00	31.00	33.00	35.00
	24575.00	31.00	33.00	35.00	37.00	39.00
	27887.50	35.00	36.00	37.00	38.00	39.00

## 3 Results and analysis

### 3.1 The airflow variation characteristics of the pneumatic conveying system

**Figure 6:**
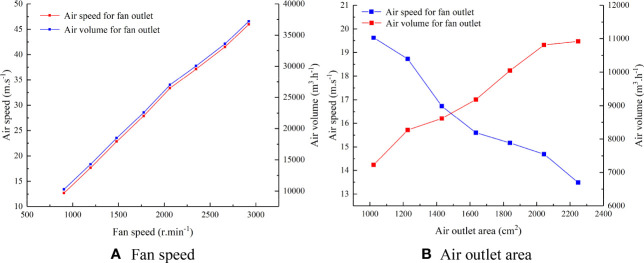
Air speed and air volume changes during the independent adjustment of the fan speed and air outlet area **(A)** Fan speed **(B)** Air outlet area.

Based on the outlet air speed and air volume data obtained in the test of the airflow variation characteristics of the pneumatic conveying system under the independent adjustment of fan speed and air outlet area, Origin software (OriginLab Corporation, USA) was used to generate plots of the changes in the outlet air speed and air volume during the independent adjustment of the fan speed and air outlet area, as shown in **Figure 6**.

**Figure 6** demonstrates that the maximum air speed and air volume of the sprayer outlet are 45.98 m/s and 37239.94 m^3^/h, respectively, and with increasing fan speed, the outlet air speed and air volume increase continuously. As the air outlet area increases, the outlet air speed continues to decrease, but the air volume continues to increase. The results indicate that changing the fan speed influences the outlet air speed and air volume in the same way, whereas changing the air outlet area effects the air speed and air volume in opposing ways. The decoupled method with constant air speed and variable air volume and the decoupled method with constant air volume and variable air speed can be achieved through the combined control of the fan speed and air outlet area to meet the air speed and air volume requirements for canopies of different fruit tree species in different growth periods.

### 3.2 Decoupled air speed and air volume adjustment model

#### 3.2.1 The air volume adjustment model with constant air speed

Through an analysis of the test data for the combined control of fan speed and outlet area, the data for the sprayer outlet air speed under different combinations of fan speed and air outlet area were obtained, as shown in **Table 3**.

**Table 3:** Air speed change at the sprayer outlet achieved by adjusting the fan speed and air outlet area.

Fan speed (r.min^-1^)	Air speed at fan outlet (m.s^-1^)						
	Air outlet area (cm^2^)						
	1022.05	1226.46	1430.87	1635.28	1839.69	2044.10	2248.51
904	13.02	12.91	12.89	12.65	12.36	12.03	11.89
1195	18.21	17.88	17.06	17.00	16.75	16.60	16.27
1480	23.35	23.01	22.34	21.97	21.26	20.94	20.68
1775	28.31	27.79	27.20	26.66	26.08	26.01	25.70
2062	34.53	33.37	31.90	31.72	30.70	30.65	30.64
2348	38.05	37.98	36.62	36.26	36.20	35.71	35.31
2662	42.62	42.02	40.92	40.31	40.18	39.52	38.51

**Figure 7:**
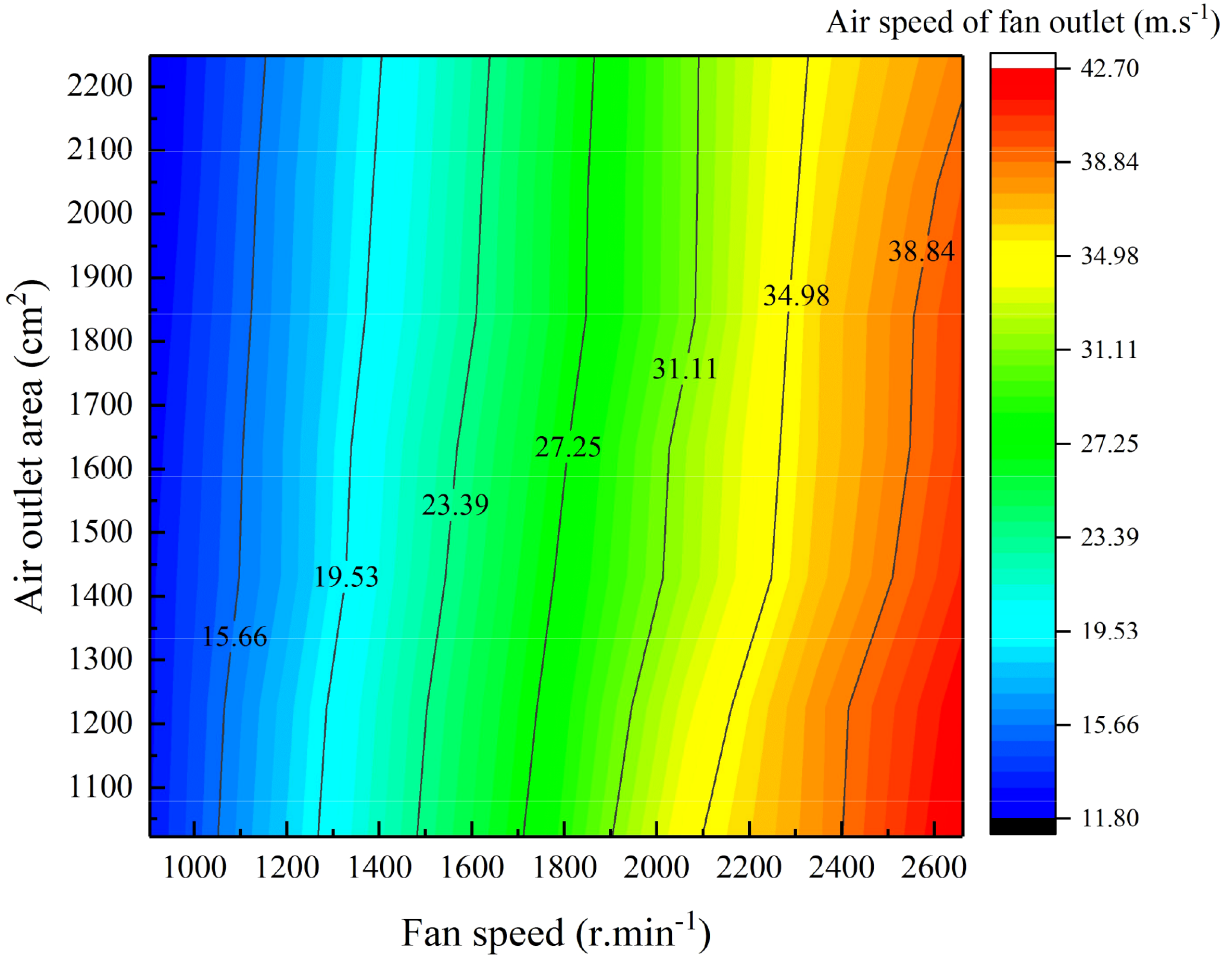
Air speed changes resulting from fan speed and air outlet area adjustments.

Origin software was used to process and analyse the obtained outlet air speed data and to fit the two-dimensional relationships between the outlet air speed and the fan speed and air outlet area, as shown in **Figure 7**.

**Figure 7** indicates that under combined fan speed and air outlet area control, the changes in the outlet air speed correspond to constant air speeds, as indicated by the contours. For each contour line, the relationship between the fan speed and the air outlet area is approximately linear. As the air outlet area increases, the fan speed increases such that the two are directly proportional, and the slope of the contour line changes in different sections. A change in the air outlet area causes a change in the outlet air volume, thereby realizing the control of the air volume at a constant air speed. The fan speed and outlet area data for the contour lines were extracted to obtain the mathematical relationships between the fan speed and the outlet area under different constant values of air speed. Based on these relationships, the mathematical equations for the fan speed and air outlet area in terms of air speed and air volume were obtained, as shown in Equation (2). The equation coefficients and *R^2^* values corresponding to different constant values of air speed are shown in **Table 4**.

**Table 4:** Coefficients of the fan speed and air volume at the fan outlet and the corresponding *R^2^* values.

Parameters	Air speed of fan outlet (m.s^-1^)						
	15.66	19.53	23.39	27.25	31.11	34.98	38.84
*a*_1_	0.229	0.318	0.377	0.362	0.463	0.518	0.605
*a_2_*	969.7	1153.3	1345.4	1587.8	1748.7	1930.2	2174.2
*R* ^2^	0.9825	0.9843	0.9798	0.9602	0.9244	0.9479	0.9573


(2) $$\left\{ \begin{array}{l} S_{OUT}=2.778\frac{AirVolume_{Bellow}}{AirSpeed_{B\text{e}llow}}\\ Speed_{Fan}=a_{1}\frac{AirVolume_{Bellow}}{AirSpeed_{B\text{e}llow}}+a_{2} \end{array}\right.$$

In the equations above, *AirVolume*_Bellow_ is the outlet air volume of the bellows (m^3^/h), *AirSpeed*_Bellow_ is the outlet air speed of the bellows (m/s), *S*_OUT_ is the air outlet area (cm^2^), *Speed*_Fan_ is the fan speed (r/min), and *a*_1_ and *a*_2_ are the coefficients of the fan speed and outlet air volume equations, respectively.

#### 3.2.2 The air speed adjustment model with constant air volume

Based on the approach in subsection 3.2.1, the outlet air speed obtained for different combinations of fan speed and outlet area adjustment was multiplied by the air outlet area at the corresponding position to obtain the outlet air volume, as shown in **Table 5**.

**Table 5:** Air volume change at the fan outlet achieved by adjusting the fan speed and air outlet area.

Fan speed (r.min^-1^)	Air volume at the fan outlet (m^3^.h^-1^)						
	Air outlet area (cm^2^)						
	1022.05	1226.46	1430.87	1635.28	1839.69	2044.10	2248.51
904	4790.82	5698.20	6642.02	7444.54	8187.30	8851.01	9623.37
1195	6700.15	7893.53	8787.10	10007.91	11090.97	12217.64	13171.13
1480	8592.93	10157.30	11509.10	12935.44	14083.09	15409.24	16737.39
1775	10416.06	12270.63	14009.98	15693.50	17272.48	19140.13	20800.90
2062	12703.06	14733.71	16434.32	18674.01	20331.78	22554.07	24798.50
2348	13999.78	16767.25	18865.65	21345.45	23975.31	26274.45	28578.11
2662	15682.31	18554.80	21075.86	23731.37	26609.80	29083.92	31170.13

**Figure 8:**
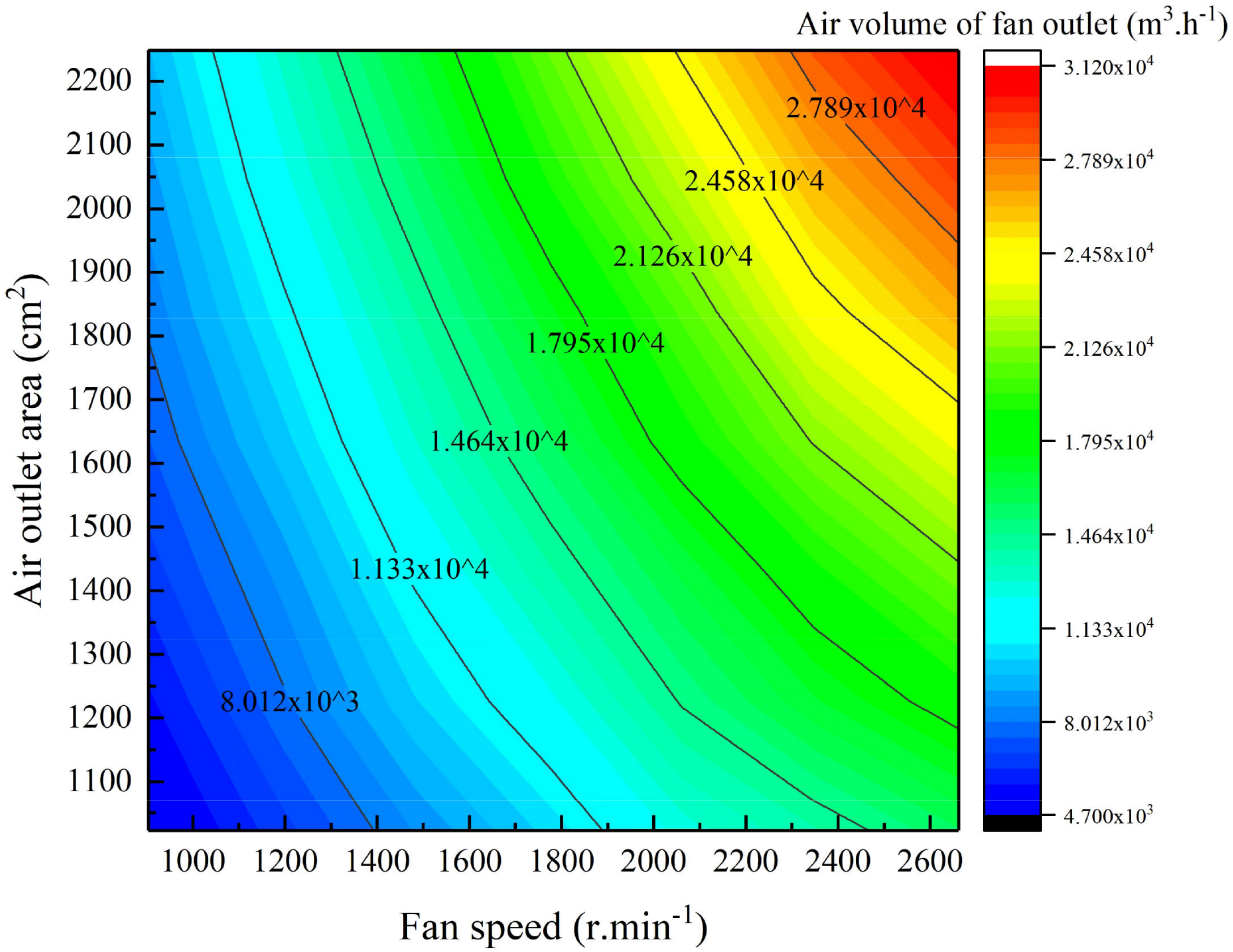
Air volume change resulting from fan speed and air outlet area adjustment.

The same data processing method as in Section 3.2.1 was used. Origin software was used to analyse the outlet air volume data and to fit the two-dimensional relationships between the outlet air volume and the fan speed and air outlet area, as shown in **Figure 8**.

**Figure 8** demonstrates that under the combined fan speed and air outlet area control, increases in the fan speed and air outlet area lead to an increase in the outlet air volume, and contour lines corresponding to constant air volumes can be observed. For each contour line, as the air outlet area increases, the fan speed decreases, and the two are inversely proportional; along the same contour line, a higher fan speed and a smaller air outlet area produce a more gradual contour change. The data processing method in Section 3.2.1 was used to establish the equations for fan speed and air outlet area based on the air speed and air volume, as shown in Equation 3. The coefficients and *R* ^2^ values of the equations for different constant values of the air volume are shown in **Table 6**.

**Table 6:** Coefficients of the fan speed and air speed at the fan outlet and the corresponding *R^2^* values.

Parameters	Air volume at the fan outlet (m^3^.h^-1^)						
	8012.5	11325.0	14637.5	17950.0	21262.5	24575.0	27887.5
*c*_1_	-1.724	-1.892	-2.499	-2.809	-2.835	-2.899	-3.398
*c_2_*	1991.2	2494.2	3217.0	3733.9	4047.9	4370.9	5034.7
*R* ^2^	0.9820	0.9614	0.9440	0.9589	0.9679	0.9709	0.9960


(3) $$\left\{ \begin{array}{l} S_{OUT}=2.778\frac{AirVolume_{Bellow}}{AirSpeed_{Bellow}}\\ Speed_{Fan}=c_{1}\frac{AirVolume_{Bellow}}{AirSpeed_{Bellow}}+c_{2} \end{array}\right.$$

where *AirVolume*_Bellow_ is the outlet air volume at the bellows (m^3^/h), *AirSpeed*_Bellow_ is the outlet air speed at the bellows (m/s), *S*_OUT_ is the air outlet area (cm^2^), *Speed*_Fan_ is the fan speed (r/min), and *c*_1_ and *c*_2_ are the coefficients of the fan speed and outlet air speed equations, respectively.

### 3.3 Verification of the control accuracy of the models

By analysing the verification test data for the decoupled air speed and air volume adjustment models, the control errors of the models under different combinations of fan speed and air outlet area were obtained, the average measurement error for each combination was determined, and the average measurement errors of all combinations were averaged to obtain the control error of each model, as shown in **Table 7**.

**Table 7:** Model validation error in air volume adjustment for a constant air speed.

Variable air volume with constant air speed		Variable air speed with constant air volume	
Constant air speed (m.s^-1^)	Average error (%)	Constant air volume (m^3^.h^-1^)	Average error (%)
15.66	1.13	8012.50	1.34
19.53	0.64	11325.00	1.02
23.39	0.57	14637.50	1.12
27.25	0.48	17950.00	1.22
31.11	0.33	21262.50	1.67
34.98	1.00	24575.00	0.50
38.84	0.38	27887.50	0.41

The air volume adjustment model with constant air speed displayed a maximum average error of 1.13% under different constant values of air speed. The air speed adjustment model with constant air volume control exhibited a maximum average error of 1.67% under different constant values of air volume. For the two control modes, the model control error was in the range of 0-2%. Therefore, the models were highly accurate.

## 4 Discussions

Most existing airflow adjustment sprayers only have a single adjustment mode, but due to the coupled relationship between the adjustments to air speed and air volume, these sprayers fail to achieve independent adjustment of air speed and air volume. The sprayer designed in this paper supports two control modes, fan speed and outlet area, which makes it possible to realize the decoupled control of air speed and air volume at the sprayer outlet. Based on the designed airflow adjustment sprayer, airflow adjustment experiments based on the independent adjustment of the fan speed and outlet area were carried out. The results showed that variable fan speed and variable outlet area had different control effects on the air speed and air volume at the sprayer outlet, and the decoupled control of the air speed and air volume was achieved through the combined control of the fan speed and outlet area; this approach provides a new research idea for the decoupled control of the air speed and air volume in orchard air-assisted spraying.

Through the combined control experiments, decoupled control models of air speed and air volume with constant air speed and variable air volume and with constant air volume and variable air speed were established. The control error of the two decoupled models was in the range of 0-2%, and the models displayed high control accuracy. However, due to the limitation of orchard spraying scenario for two decoupled models, the verification tests of the models were only carried out in the laboratory. The decoupled control models were for the subsequent research and development of the airflow online on-demand adjustment system based on orchard tree target characteristics (canopy volume, leaf area, etc.). The adjustment values of air speed and air volume are calculated according to the orchard tree target characteristics, and they are the values of air speed and air volume from generation to propagation to canopy position. However, the response delay of the airflow adjustment device itself, the propagation time of airflow from fan outlet to fruit tree canopy and the influence of natural wind on airflow propagation trajectory will have an impact on the control accuracy of airflow online on-demand adjustment system. In the future, orchard verification tests design for the decoupled control models, the above factors should be considered, and the decoupled control model of air speed and air volume proposed in this paper should be evaluated more reasonably combined with practical orchard spraying scenario.

The two decoupled models can meet the different requirements for air speed and air volume of the differentiated canopies. When the density of the fruit tree canopy does not change significantly but the canopy volume does change significantly, it is necessary to adjust the air volume at a constant air speed to achieve the best pesticide application. For example, during the period from germination to the first leaf stage for grapevines and standardized densely planted dwarf apple trees, the density of canopy branches and leaves does not change significantly, but the canopy volume changes considerably. However, when the volume of the fruit tree canopy does not change significantly but the density of the fruit tree canopy does change significantly, orchard air-assisted spraying requires a constant outlet air volume and a variable air speed. For example, the canopy contours of apple trees and peach trees are formed during the period from the complete growth of new leaves to the fruiting period; during the leaf growth period of apple trees and peach trees, the canopy volume changes little, but the canopy leaf area changes substantially. The decoupled control model provides a mathematical support for overcoming the key bottleneck of on-demand airflow control during air-assisted spraying and promoting the application of precision spraying technology for orchards. The research team has broken through the online detection models of tree canopy volume and leaf area (Gu et al., 2022; Wang et al., 2023), and the airflow online on-demand adjustment system based on orchard tree target characteristics will be developed in the future.

## 5 Conclusions

In this study, an innovative airflow adjustment sprayer that supports the independent adjustment of fan speed (0-2940 r/min) and air outlet area (1022.05-2248.51 cm^2^) is developed, and the control ranges of the maximum air speed and air volume at the air outlet of the sprayer are 45.98 m/s and 37239.94 m^3^/h, respectively. Based on the sprayer, independent control tests of fan speed and air outlet area were carried out. The results showed that changes in the fan speed and air outlet area have opposing effects on the air speed and air volume, and the decoupled control of the air speed and air volume can be achieved through the combined control of the fan speed and air outlet area, thus providing a new research concept for the decoupled control of the air speed and air volume at the sprayer outlet.

Decoupled airflow control tests were carried out by adjusting the fan speed and outlet area to control the air speed and air volume at the sprayer outlet in different ranges of combinations. Two decoupled air speed and air volume adjustment models (one with constant air speed and variable air volume and the other with constant air volume and variable air speed) were established by the contour line fitting of the outlet air speed and air volume data obtained for different combinations of fan speed and air outlet area. The model validation test results show that the air volume adjustment model with constant air speed had a maximum mean error of 1.13% and that the air speed adjustment model with constant air volume had a maximum mean error of 1.67%. The model with constant air speed and variable air volume is suitable for spraying scenarios in which the density of the fruit tree canopy does not change significantly but the canopy volume does change significantly, and the model with constant air volume and variable air speed is suitable for spraying scenarios in which the canopy volume does not change significantly but the density of the fruit tree canopy does change significantly. The results of this study provide theoretical and methodological support for research on and the development of airflow adjustment systems and sprayers for orchard air-assisted spraying.

## Data availability statement

The original contributions presented in the study are included in the article/supplementary material. Further inquiries can be directed to the corresponding authors.

## Author contributions

HD, CZ and LC: conceptualisation, validation, investigation and methodology. HD, SY and YZ: software and visualisation. HD, YZ and SY: formal analysis. HD, CZ and CG: data curation. GC and CZ: resources and supervision. HD and CZ: writing—original draft preparation. HD, CZ and LC: writing—review and editing and funding acquisition. CZ: project administration. All authors contributed to the article and approved the submitted version.
